# Presence of β-Lactamase-producing Enterobacterales and *Salmonella* Isolates in Marine Mammals

**DOI:** 10.3390/ijms22115905

**Published:** 2021-05-31

**Authors:** Olivia M. Grünzweil, Lauren Palmer, Adriana Cabal, Michael P. Szostak, Werner Ruppitsch, Christian Kornschober, Maciej Korus, Dusan Misic, Tanja Bernreiter-Hofer, Anna D. J. Korath, Andrea T. Feßler, Franz Allerberger, Stefan Schwarz, Joachim Spergser, Elke Müller, Sascha D. Braun, Stefan Monecke, Ralf Ehricht, Chris Walzer, Hrvoje Smodlaka, Igor Loncaric

**Affiliations:** 1 Institute of Microbiology, University of Veterinary Medicine, 1210 Vienna, Austria; 01245128@students.vetmeduni.ac.at (O.M.G.); michael.szostak@vetmeduni.ac.at (M.P.S.); tanja.bernreiter-hofer@vetmeduni.ac.at (T.B.-H.); koratha@staff.vetmeduni.ac.at (A.D.J.K.); joachim.spergser@vetmeduni.ac.at (J.S.); 2 Marine Mammal Care Center, Los Angeles, CA 90731, USA; lpalmer@marinemammalcare.org; 3 Austrian Agency for Health and Food Safety (AGES), Institute of Medical Microbiology and Hygiene, 1090 Vienna, Austria; adriana.cabal-rosel@ages.at (A.C.); werner.ruppitsch@ages.at (W.R.); franz.allerberger@ages.at (F.A.); 4 Austrian Agency for Health and Food Safety (AGES), National Reference Centre for Salmonella, 8010 Graz, Austria; christian.kornschober@ages.at; 5 Department of Functional Food Products Development, Faculty of Biotechnology and Food Science, Wroclaw University of Environmental and Life Sciences, 51-630 Wroclaw, Poland; maciej.korus@upwr.edu.pl (M.K.); dusan.misic@upwr.edu.pl (D.M.); 6 Department for Farm Animals and Veterinary Public Health, University Clinic for Swine, University of Veterinary Medicine, 1210 Vienna, Austria; 7 Centre for Infection Medicine, Department of Veterinary Medicine, Institute of Microbiology and Epizootics, Freie Universität Berlin, 14163 Berlin, Germany; andrea.fessler@fu-berlin.de (A.T.F.); stefan.schwarz@fu-berlin.de (S.S.); 8 Leibniz Institute of Photonic Technology (IPHT), 07745 Jena, Germany; elke.mueller@leibniz-ipht.de (E.M.); sascha.braun@leibniz-ipht.de (S.D.B.); stefan.monecke@leibniz-ipht.de (S.M.); ralf.ehricht@leibniz-ipht.de (R.E.); 9 InfectoGnostics Research Campus, 07743 Jena, Germany; 10 Institute for Medical Microbiology and Hygiene, Technical University of Dresden, 01307 Dresden, Germany; 11 Institute of Physical Chemistry, Friedrich Schiller University Jena, 07743 Jena, Germany; 12 Research Institute of Wildlife Ecology, University of Veterinary Medicine, 1160 Vienna, Austria; cwalzer@wcs.org; 13 Health Program, Wildlife Conservation Society, Bronx, New York City, NY 10460, USA; 14 College of Veterinary Medicine, Western University of Health Sciences, Pomona, CA 91766-1854, USA; hsmodlaka@westernu.edu

**Keywords:** antimicrobial resistance, ESBL/AmpC, wildlife, *E. coli*, *K. pneumoniae*

## Abstract

Marine mammals have been described as sentinels of the health of marine ecosystems. Therefore, the aim of this study was to investigate (i) the presence of extended-spectrum β-lactamase (ESBL)- and AmpC-producing Enterobacterales, which comprise several bacterial families important to the healthcare sector, as well as (ii) the presence of *Salmonella* in these coastal animals. The antimicrobial resistance pheno- and genotypes, as well as biocide susceptibility of Enterobacterales isolated from stranded marine mammals, were determined prior to their rehabilitation. All *E. coli* isolates (*n* = 27) were screened for virulence genes via DNA-based microarray, and twelve selected *E. coli* isolates were analyzed by whole-genome sequencing. Seventy-one percent of the Enterobacterales isolates exhibited a multidrug-resistant (MDR) pheno- and genotype. The gene *bla*_CMY_ (*n* = 51) was the predominant β-lactamase gene. In addition, *bla*_TEM-1_ (*n* = 38), *bla*_SHV-33_ (*n* = 8), *bla*_CTX-M-15_ (*n* = 7), *bla*_OXA-1_ (*n* = 7), *bla*_SHV-11_ (*n* = 3), and *bla*_DHA-1_ (*n* = 2) were detected. The most prevalent non-β-lactamase genes were *sul2* (*n* = 38), *strA* (*n* = 34), *strB* (*n* = 34), and *tet*(A) (*n* = 34). *Escherichia coli* isolates belonging to the pandemic sequence types (STs) ST38, ST167, and ST648 were identified. Among *Salmonella* isolates (*n* = 18), *S*. Havana was the most prevalent serotype. The present study revealed a high prevalence of MDR bacteria and the presence of pandemic high-risk clones, both of which are indicators of anthropogenic antimicrobial pollution, in marine mammals.

## 1. Introduction

Antimicrobial resistance (AMR) in bacteria is an emerging public health concern worldwide. Particularly, AMR among Enterobacterales is globally widespread and constitutes a public health threat due to a high prevalence of multidrug-resistant (MDR) strains [1,2]. MDR Gram-negative bacteria may complicate antimicrobial therapy. In addition to resistance classification of bacteria (multidrug resistance (MDR), extensive drug resistance (XDR), and pandrug resistance (PDR)) in the last decade, several novel classification criteria that correlate well with clinical outcomes were introduced (e.g., difficult-to-treat resistance (DTR)). This additional classification criterion will lead to significant benefits in assessing the significance of bacterial resistance in therapeutics [3,4].

Extended-spectrum beta (β)-lactamases (ESBL) and AmpC β-lactamases (AmpC) are the most common enzymes imparting broad-spectrum cephalosporin resistance in Enterobacterales [5,6]. Many enterobacterial species, e.g., *Citrobacter* spp. and *Enterobacter* spp., harbor inducible chromosomally-encoded AmpC β-lactamases, whose production can be triggered by exposure to β-lactam antibiotics, especially strong inducers (benzylpenicillin, ampicillin, amoxicillin, and cephalosporins (cefazolin and cephalothin)) [6,7,8]. In contrast, β-lactamase (*bla*) genes of *Escherichia* (*E*.) *coli*, *Klebsiella* spp., and *Salmonella* spp. are mainly carried on mobile genetic elements, such as plasmids, which are considered to be primarily responsible for the rapid spread of β-lactam resistances among these Enterobacterales [6,8,9].

Despite the intensively studied occurrence of AMR in bacteria from humans and domestic animals, there is a lack of knowledge about the distribution of AMR among bacteria from marine mammals. The Northeastern Pacific Ocean is a rich habitat that sustains a massive amount of phytoplankton, zooplankton, krill, and fish, all of which support a diverse and abundant community of more than 30 species of marine mammals [10]. Previous observations indicate that the marine environment may act as a reservoir of antimicrobial resistance genes [11,12,13,14]. Different mechanisms can lead to the presence of antimicrobial-resistant bacteria in the oceans: first, anthropogenic antimicrobial-containing runoff, challenging native bacteria to become resistant; second, coastal runoff of already resistant bacteria from terrestrial sources; and third—although less important in this context—antimicrobial production by marine microorganisms [11,15].

Wildlife sampled in habitats impacted by livestock production, solid waste disposal, wastewater treatment, and other anthropogenic influences have been found to result in a higher prevalence of antimicrobial-resistant bacteria compared to less influenced habitats [16]. The global use of antimicrobial agents reaching the oceans via municipal sewage, commercial fish farms, and hospitals [17,18] is likely to increase AMR in bacteria from marine mammals and marine microbial communities [19]. It has been reported that wildlife, in general, might act as an indicator of the burden of resistance within a local environment and could help to identify sources of anthropogenic contamination with antimicrobial resistance determinants [16,20,21,22]. For these reasons, marine mammals, such as pinnipeds and dolphins residing in coastal waters, have been described as sentinels of the health of coastal marine ecosystems and, consequently, also the health of humans living in or visiting these areas [23,24,25]. Nevertheless, information on AMR in bacteria from marine mammals remains scarce [19,26,27,28,29,30].

The present study aimed to determine the occurrence and to characterize ESBL- and AmpC β-lactamase-producing Enterobacterales, including non-resistant *Salmonella* spp., in stranded marine mammals temporarily housed in the Marine Mammal Care Center Los Angeles (MMCCLA), California. Included in this study were California sea lions (*Zalophus californianus*), northern fur seals (*Callorhinus ursinus*) and one Guadalupe fur seal (*Arctocephalus townsendi*) representing Otariid seals, northern elephant seals (*Mirounga angustirostris*) and harbor seals (*Phoca vitulina*) representing Phocid seals, and a long-beaked common dolphin (*Delphinus capensis*) and a pygmy sperm whale (*Kogia breviceps*) representing Cetaceans.

## 2. Results

### 2.1. β-Lactamase Producing Isolates, Species Identification and Phenotypic Resistance 

Out of a total of 283 samples, 62 β-lactamase-producing Enterobacterales were obtained and identified as *E. coli* (*n* = 27), *Klebsiella* (*K*.) *pneumoniae* (*n* = 12), *Citrobacter* (*C*.) *freundii* complex and *C. koseri* (each *n* = 9), *Enterobacter* (*En*.) *cloacae* complex and *En. cancerogenus* (each *n* = 1), *Lelliottia* (*L*.) *amnigena* (*n* = 2), and *Proteus* (*P.*) *mirabilis* (*n* = 1). Most of them (*n* = 51) displayed an AmpC phenotype, whereas all *Citrobacter* and *Enterobacter* isolates were identified as stably de-repressed AmpC-producers. Five *E. coli* isolates (IDs 41, 68, 183, 209, and 234) displayed an ESBL phenotype, and two *E. coli* (IDs 21, 90) and the two *L. amnigena* isolates (IDs 130 and 132) displayed both AmpC and ESBL phenotypes (Table 1 and Table 2).

In addition to β-lactam resistance, resistance to trimethoprim-sulfamethoxazole (*n* = 47; 75.81%), tetracycline (*n* = 42; 67.74%), chloramphenicol (*n* = 32; 51.61%), gentamicin (*n* = 30; 48.39%), tobramycin (*n* = 25; 40.32%), ciprofloxacin (*n* = 12; 19.35%), and fosfomycin (*n* = 9; 14.52%) were expressed by the enterobacterial isolates. Forty-four isolates exhibited a multidrug-resistant phenotype [31]. All of the isolates were susceptible to carbapenems.

Seventeen of the 18 *Salmonella* isolates obtained in this study were susceptible to all tested antimicrobial agents. One *Salmonella* isolate (ID 42 S.) displayed an AmpC phenotype, but no further resistance to non-β-lactam antibiotics was observed.

Biocide susceptibility testing revealed elevated minimal inhibitory concentration (MIC) values for chlorhexidine in 45 out of the 62 enterobacterial isolates (79%) and in all *Salmonella* isolates (18/18). MIC values for isopropanol were elevated in 23 Enterobacterales (31%) and two *Salmonella* isolates. One *Salmonella* isolate (ID 245 S.) exhibited an elevated MIC value for glutardialdehyde. For all but one isolate (ID 230b; MIC 0.000125%), the MICs of benzalkonium chloride ranged between 0.001% and 0.002% (Supplementary Table S1).

### 2.2. Characterization of Genotypic Resistance

Within the group of β-lactamase genes, *bla*_CMY_ (*n* = 51) was the most prevalent and was detected solely (*n* = 13) or together with *bla*_CTX-M_, *bla*_OXA_, *bla*_SHV_, and/or *bla*_TEM_. All sequenced *bla*_CMY_ genes were identified as *bla*_CMY-2_, except one *E. coli* isolate (ID 171) harboring *bla*_CMY-4_. Sequencing of *bla*_CMY_ was not carried out in intrinsically AmpC-producing Enterobacterales, such as *Citrobacter* spp. and *Enterobacter* spp. The gene *bla*_TEM_ was detected in 38 isolates—solely (*n* = 1) or together with *bla*_CMY_, *bla*_CTX-M_, *bla*_OXA,_ and/or *bla*_SHV_. All *bla*_TEM_ genes were identified as *bla*_TEM-1_. *bla*_SHV_ genes were detected in 13 different isolates. The broad-spectrum β-lactamase genes *bla*_SHV-11_ (*n* = 3) and *bla*_SHV-33_ (*n* = 8) were detected in 11 of the 12 *K. pneumoniae* isolates, together with *bla*_TEM_ and *bla*_CMY_ (n = 8) or together with *bla*_TEM_, *bla*_CMY,_ and *bla*_OXA_ (*n* = 3). The ESBL gene *bla*_SHV-12_ (*n* = 2) was harbored by the two *L. amnigena* isolates (IDs 130, 132), which also carried the gene *bla*_DHA-1_. The genes *bla*_CTX-M-15_ and *bla*_OXA-1_ were observed in seven isolates, respectively (Table 1 and Table 2). 

The gene *bla*_CMY-2_ was detected in the *Salmonella* isolate 42 S.

In two of the *E. coli* isolates (IDs 3 and 202) displaying an AmpC phenotype, no β-lactamase gene was detected, but mutations in the promoter and attenuator region of the chromosomal *ampC* gene were found at positions –18 (G→A), –1 (C→T) and +58 (C→T) in *E. coli* isolate 3 and at position –32 (T→A) in *E. coli* isolate 202. Further, mutations were found at position –18 (G→A), –1 (C→T), and +58 (C→T) of the *ampC* gene of *E. coli* isolates 8/2 and 21, at positions –18 (G→A) and –1 (C→T) in *E. coli* 192/2, at position +58 (C→T) in *E. coli* 17, and at position –28 (G→A) of the *ampC* gene of *E. coli* isolates 197/1 and 224a.

The most prevalent non-β-lactamase genes detected were *sul2* (*n* = 38), *strA* (*n* = 34), *strB* (*n* = 34), *tet*(A) (*n* = 34), and *sul1* (*n* = 33).

The analysis of the quinolone resistance-determining regions (QRDR) of *gyrA* and *parC* revealed mutations that resulted in amino acid exchanges at positions 83 (Ser→Leu) and 87 (Asp→Asn) in GyrA of five ciprofloxacin-resistant isolates and at position 80 (Ser→Ile) in ParC of six ciprofloxacin-resistant isolates.

Even though phenotypic resistance to tetracycline was present, none of the tested *tet* genes were detected in *C. freundii* complex isolate 224b.

Antimicrobial resistance phenotypes and genes are summarized in Table 1 and Table 2.

### 2.3. Molecular Typing Methods

The most common *E. coli* phylogenetic group was A (*n* = 8). Four *E. coli* isolates each were assigned to the groups B1, B2, D, and F, while three isolates represented phylogroup E (Table 1 and Table 3).

**Figure 1:**
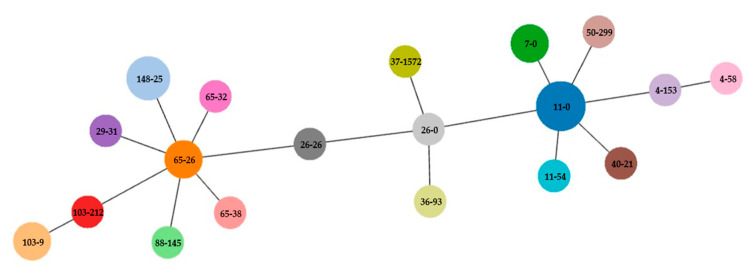
Clonal phylogeny characterized by goeBURST diagram of the CH clonotyping data set of *E. coli* isolates. An eBURST diagram was calculated using PHYLOViZ with the goeBURST algorithm. *E. coli* isolates were grouped according to their CH profiles.

The *fumC* and *fimH* (CH) typing divided the *E. coli* isolates into 19 distinct CH clonotypes and revealed clonal relatedness of *E. coli* isolates 147a and 149a (CH7-0); 3 and 8/2 (CH65-26); 117 and 202 (CH103-9); 53/1, 92 and 158 (CH148-25); and 68, 156, 183, and 230b (CH11-0) (Figure 1).

Multilocus sequence typing (MLST) of *Klebsiella pneumoniae* identified two different types among the tested isolates: ST466 (*n* = 9) and ST406 (*n* = 3).

### 2.4. Characterization of E. coli Virulence Genes

The most common *E. coli* virulence genes determined via microarray were *fimH* (15/15), *iucD* and *papC* (each 5/15), *astA* (4/15), and *cnf1* (3/15) (Table 1). The gene *hlyA* was detected in one isolate (ID 17) accompanied by *cnf1*. The genes *iucD* and *papC* were detected simultaneously in five *E. coli* isolates. Those five *E. coli* isolates (IDs 21, 53/1, 92, 156, and 158) were assigned to extraintestinal pathogenic *E. coli* (ExPEC)/uropathogenic *E. coli* (UPEC) pathotype due to the presence of the virulence genes *fimH*, *iucD,* and *papC* [32].

### 2.5. Whole-Genome Sequencing (WGS) of Selected E. coli Isolates

Of the 12 whole-genome sequenced *E. coli* isolates, eleven originated from California sea lions and one from a northern elephant seal. Three isolates (IDs 68, 183, and 230b) belonged to the sequence type (ST)167 and clustered together by core genome multilocus sequence-based typing (cgMLST), which suggested clonal relatedness between them. Other sequence types obtained were ST38, ST349, ST372, ST484, ST648, ST963, ST1431, ST4957, and ST5748. E. coli predicted serotype (somatic O and flagellar H antigens) was clearly determined in one isolate (ID 192/2) as O9:H10 (Table 3).

The multidrug transporter gene *mdfA*, which confers resistance to a diverse group of biocides and antimicrobial agents [33], was detected in all 12 isolates analyzed by WGS. 

The genes associated with biocide resistance *emrK* (*n* = 12), *mdtE* (*n* = 12), *mdtF* (*n* = 12), *tolC* (*n* = 12), *acrF* (*n* = 12), *acrE* (*n* = 9), and *emrE* (*n* = 5), which impart decreased susceptibility to benzalkonium chloride, were identified. Furthermore, the genes *cpxA* (*n* = 10) and *qacEΔ1* (*n* = 5), conferring decreased susceptibility to benzalkonium chloride and chlorhexidine, were detected. Biocide susceptibility phenotypes and genes associated with elevated biocide MICs are summarized in Supplementary Table S1.

WGS revealed that different virulence genes were present in the *E. coli* isolates with the tellurite resistance gene *terC* (*n* = 12), the increased serum survival gene *iss*, the serum-resistance associated gene *traT*, the yersiniabactin encoding gene *irp2*, the yersiniabactin receptor encoding gene *fyuA*, and the iron and manganese transport gene *sitA* (each *n* = 8) being predominant (Table 3).

The determination of plasmids via the PlasmidFinder software revealed that the predominant plasmid was IncFIB (*n* = 7). In the *E. coli* isolate 209, the *bla*_TEM-1B_ gene was carried by an IncI1-Iα plasmid (Supplementary Tables S2 and S3).

The mlplasmids analyses predicted that the majority of the antimicrobial resistance genes might be plasmid-encoded, whereas most virulence genes appeared to be chromosomally-encoded (Supplementary Tables S3 and S4). The *bla*_CTX-M-15_ gene might be located in the chromosomal DNA of two isolates (IDs 209 and 234). In *E. coli* 68 and 183, a prediction for the location of the *bla*_CTX-M-15_ gene could not be made. A chromosomally-encoded *bla*_CMY-2_ gene might have been obtained in *E. coli* 206 and 230a, and a chromosomally-encoded *bla*_CMY-4_ in *E. coli* 171.

### 2.6. Characterization of Salmonella Isolates

The most common serotypes among the 18 analyzed *Salmonella* isolates were *Salmonella* (*S.*) Havana (antigenic formula: 1,13,23 : f,g : -) (*n* = 8) and *S.* Reading (antigenic formula: 1,4,5,12 : e,h : 1,5) (*n* = 3). Furthermore, *S*. Albany (*n* = 2), *S*. Newport (*n* = 2), *S*. Saintpaul (*n* = 2), and *S*. Braenderup (*n* = 1) were identified. (Table 4).

In all *Salmonella* isolates (*n* = 18) the transcriptional regulator gene *hilA* and *Salmonella* enterotoxin gene *stn* were detected. The virulence gene *tviA* was not found.

## 3. Discussion

In this study, we investigated the phenotypic and genotypic characteristics of ESBL- and AmpC-producing Enterobacterales, as well as the presence of *Salmonella* isolates in stranded marine mammals prior to their rehabilitation. The majority of the investigated β-lactamase-producing Enterobacterales exhibited an AmpC phenotype, an ESBL phenotype was observed in five *E. coli* isolates, and both AmpC and ESBL phenotypes were present in two *E. coli* and two *L. amnigena* isolates. In the scope of this study, three types of AmpC resistance mechanisms have been identified: plasmid-mediated AmpC (pAmpC) genes, overexpression of chromosomal AmpC in *E. coli*, and de-repressed AmpC in *Citrobacter* spp. and *Enterobacter* spp. AmpC-producers have been reported as particularly common in North America, whereas in Europe, β-lactamases are primarily represented by ESBLs [34]. Among the *bla* genes investigated in this study, the pAmpC gene *bla*_CMY_ was detected most frequently. It was in all *K. pneumoniae* and *P. mirabilis* isolates, in one *Salmonella* isolate, and in 18 *E. coli* isolates identified as *bla*_CMY-2_, which encodes the most prevalent pAmpC enzyme in humans, livestock, and companion animals worldwide [7,9]. Moreover, *bla*_CMY-2_ has been detected in different wildlife species [35,36,37,38]. In two *E. coli* isolates (IDs 206 and 230a), *bla*_CMY-2_ is predicted to be encoded chromosomally, which was described in 2015 for the first time in *E. coli* [39]. In addition, we obtained a high number of isolates exhibiting *bla*_TEM-1_ (*n* = 38) and, less frequently, *bla*_OXA-1_ (*n* = 7). Both *bla* genes were recently detected in *E. coli* isolates from harbor seals and grey seals (*Halichoerus grypus*) in Ireland [40], in which, in contrast to the findings of the present study, *bla*_OXA-1_ was more frequently represented than *bla*_TEM-1_. All but one (ID 160) *K. pneumoniae* isolates carried a *bla*_SHV_ gene. The broad-spectrum β-lactamase genes *bla*_SHV-11_ and *bla*_SHV-33_ were detected exclusively in ST466 *K. pneumoniae* and ST405 *K. pneumoniae*, respectively. Chaves et al. were the first to detect the novel SHV-type variant SHV-33 in *K. pneumoniae,* and they hypothesized that the ancestor of SHV-1 β-lactamase originated from the *K. pneumoniae* chromosomal DNA [41]. Furthermore, the pAmpC gene *bla*_DHA-1_, which was detected in the two *L. amnigena* isolates (IDs 130 and 132), was previously also obtained in a *K. pneumoniae* isolate originating from a free-living mouflon in Austria [42].

Besides resistance to β-lactam antibiotics, resistance to other antibiotic classes was common among the investigated Enterobacterales. In total, 71% of the isolates exhibited a MDR phenotype and genotype. The study on *E. coli* in seals in Ireland determined a high proportion of MDR bacteria (66.6%) in the animals tested [40]. Already in the 1990s, AMR in Enterobacterales appeared widespread among Californian pinnipeds [26]. Wallace et al. [19] observed an increase of AMR in bacteria of stranded pinnipeds of the Northwest Atlantic over a period of 6 years (2004–2010). *E. coli* especially displayed a considerable increase in resistance to β-lactams, sulfonamides, and aminoglycosides, while *Klebsiella* spp. exhibited an increase in resistance to aminoglycosides and fluoroquinolones [19].

Biocide susceptibility testing revealed a high proportion of elevated MIC values for chlorhexidine (79%) and isopropanol (31%). Information regarding biocide susceptibility of Enterobacterales isolated from wildlife is scarce. A recent study on *K. pneumoniae* from Californian pinnipeds investigated environmental persistence, as well as disinfectant susceptibility, of these bacteria [43]. Commonly recommended concentrations of chlorine and hydrogen peroxide turned out to be ineffective in eradication of the biofilm formed by hypermucoviscous *K. pneumoniae*, whereas ethanol and chloramine-T effectively eradicated *K. pneumoniae*, irrespective of whether formed in biofilm or not [43].

Furthermore, sublethal concentrations of biocidal agents, in particular, benzalkonium chloride, chlorhexidine, and triclosan, may enhance antimicrobial tolerance and resistance in Gram-negative bacteria [44,45].

Among the *E. coli* isolates, six different phylogenetic groups were determined, with the most common being phylogroup A (*n* = 8), described as being widespread among human commensal *E. coli* strains [46]. Contrary to our findings, investigations in Australia revealed the human-associated phylogroups B2 and D as the predominating *E. coli* phylotypes in pinnipeds [47,48,49].

cgMLST revealed that three *E. coli* clones (IDs 68, 183, and 230b) belonged to A-O_NT_:H9-ST167-CH11-0. These isolates were obtained from different individuals over a time-span of several months and exhibited in part highly diverse resistance pheno- and genotypes. This could indicate that these clones were already circulating for an extended period of time within the tested population of marine mammals and in their environment. Therefore, several resistance gene exchanges with other bacteria could have taken place. Related investigations have been reported regarding interspecies transmission of an *E. coli* ST410 clone between wildlife, humans, companion animals and the environment within several years in Berlin, Germany [50]. 

*E. coli* ST167 is considered a pandemic clone of significant public health concern due to its ESBL-producing strains [9]. Rising numbers of ST167 strains carrying the *bla*_NDM_ carbapenemase gene were being reported and highlighted the threat emanating from this clone [51,52,53]. Guenther et al. were the first to report occurrence of ST167 ESBL-*E. coli* in wildlife [54]. Moreover, *E. coli* ST167 was detected in cloacal samples of silver gulls in Australia [55]. 

The MDR AmpC-producing *E. coli* isolate 197/1 belonged to ST648, a pandemic high risk clone combining MDR and high-level virulence [56], which has already been isolated from wild birds in Germany [50,57] and zoo animals in Israel [58].

*E. coli* isolate 209 was identified as ST38, a prevalent human clinical pathogen that has also been reported in wild birds of prey in Mongolia [59], rats in Africa [60], and silver gulls in Australia [61]. Furthermore, ST38 has had an entry in the Enterobase Escherichia/Shigella Database, whose corresponding isolate originated from a sea lion in Ecuador, South America (https://enterobase.warwick.ac.uk/species/index/ecoli, accessed on 21 March 2021) [62]. Both *E. coli* ST38 and ST648 are known as emerging ExPEC worldwide [63,64].

*E. coli* ST349, ST963, and ST1431 have also been detected in samples from wild birds [65], and an ST372 *E. coli*, isolated from a straw-coloured fruit bat in the Republic of Congo, has previously been detected and classified as ExPEC [66]. Additionally, *E. coli* ST372 has had an entry in the aforementioned database and the respective originated from a common bottlenose dolphin (*Tursiops truncatus*) in Mexico. There are no reports describing the isolation of *E. coli* ST484, ST4957, and ST5748 from wildlife to the authors knowledge. 

An alarmingly high prevalence of different *E. coli* virulence genes was found in the isolates investigated. Most of these virulence genes could be put into context with ExPEC and/or UPEC, e.g., *cnf1, fimH, fyuA, iroN, iss, iucD, iutA,* kpsM*II, papA, papC, sitA,* and *traT* [67,68]. *E. coli* virulence genes associated with ExPEC were also detected in seals in Ireland [40] and in marine sediments from coasts of the Adriatic Sea [69].

*K. pneumoniae* is a nosocomial pathogen with increasing MDR rates and global dissemination of high risk clones [70]. Although several studies have previously focused on *K. pneumoniae* in farm and companion animals [71,72,73,74], information about this pathogen in wildlife is still scarce [42,55,75,76,77,78]. Nevertheless, there are some reports about the isolation of *K. pneumoniae* from marine mammals along the U.S. Pacific Coast [19,79,80,81]. Among the *K. pneumoniae* isolates in the present study, two distinct sequence types were obtained: ST405 (*n* = 3) and ST466 (*n* = 9). The three *K. pneumoniae* isolates (IDs 161, 173, and 175) belonging to ST405 displayed indistinguishable resistance pheno- and genotypes and exhibited 17 different resistance genes each. This might indicate the importance of this clone in the investigated environment. *K. pneumoniae* ST405 strains isolated from humans have been reported worldwide to harbor ESBL and carbapenemase genes [82,83,84,85]. However, reports about *K. pneumoniae* ST466 are scarce. One study revealed a context of *K. pneumoniae* ST466 harboring *bla*_CTX-M-15_ with causing neonatal sepsis [86].

*Salmonella* spp. have been previously isolated from marine mammals in California [87,88,89,90]. Within the scope of the present study, 18 *Salmonella* isolates belonging to six different serotypes (*S*. Havana, *S*. Reading, *S*. Albany, *S*. Newport, *S*. Saintpaul, *S*. Braenderup) were obtained, and one isolate exhibited AMR. Our findings regarding *Salmonella* are in general accordance with those of Stoddard et al. [82]. They investigated the prevalence and antimicrobial susceptibility of *Salmonella* spp. in northern elephant seals on the Californian coast. They further determined a higher prevalence and higher antimicrobial resistance rates of *Salmonella* in stranded seals than in young pinnipeds that had never entered the seawater on their natal beaches, presumably because stranded animals are more susceptible to infection by pathogens in the marine environment from terrestrial sources [87]. Furthermore, another study on *Salmonella* in Californian seals revealed that the amount of precipitation within the immediate pre-sampling period correlates positively with the presence of fecal *Salmonella* spp. [90].

As previously reported, marine mammals living in coastal habitats as predatory species with long lifespans can serve as sentinels of the health of the ocean and its inhabitants [24,91]. The high burden of AMR bacteria among marine mammals reported in this study might be the result of increasing anthropogenic pollution of the marine environment with antimicrobial agents, biocides, and already resistant bacteria. To get more detailed insights into the broad field of possible environmental risk factors and to identify notable sources of contamination, further comparative studies are needed.

## 4. Materials and Methods

### 4.1. Bacterial Isolates

Samples were collected from April 2019 to December 2020 upon admission of the stranded marine mammals to the Marine Mammal Care Center Los Angeles (MMCCLA) as part of diagnostic health evaluation. In all but four cases, samples were collected prior to any antimicrobial treatment. Overall, 145 rectal swabs, 130 oral swabs, four wound swabs, two swabs of a blow hole, and two swabs of the nictitating membrane were sampled from 148 individual animals, including 122 California sea lions (*Zalophus californianus*), 15 northern elephant seals (*Mirounga angustirostris*), six harbor seals (*Phoca vitulina*), two northern fur seals (*Callorhinus ursinus*), one Guadalupe fur seal (*Arctocephalus townsendi*), one long-beaked common dolphin (*Delphinus capensis*), and one pygmy sperm whale (*Kogia breviceps*).

Each sample was preincubated at 37 °C overnight in buffered peptone water (BPW) (Merck, Germany). For selective isolation of β-lactamase-producing Enterobacterales, 200 µL of the incubated sample were cultured at 37 °C overnight in BPW supplemented with cefotaxime (1 mg/L) (BPWCTX) and then cultivated at 37 °C overnight on MacConkey agar (Oxoid, Basingstoke, United Kingdom) supplemented with cefotaxime (1 mg/L) (MacCTX). In parallel, another 200 µL of the preincubated sample were cultured at 42 °C overnight in Rappaport–Vassiliadis-broth (RV) (Oxoid, Basingstoke, UK) and in Selenit-broth (Merck, Darmstadt, Germany) and then cultivated at 37 °C on BD™ XLD Agar (Xylose-Lysine-Desoxycholate Agar) (BD, Heidelberg, Germany) for selective isolation of *Salmonella* sp. After incubation on MacCTX, one colony representing a distinct colony morphotype was regrown on BD MacConkey II Agar (MC) at 37 °C overnight. After incubation on XLD, the respective colonies of *Salmonella* isolates showed the typical colony appearance of *Salmonella* and were selected for further characterization. Colonies selected from MacCTX and XLD were identified to the species level by matrix-assisted laser desorption/ionization-time-of-flight (MALDI-TOF) mass spectrometry (Bruker Daltonik, Heidelberg, Germany). Isolates, which grew on MacCTX and were confirmed as belonging to Enterobacterales, were examined for ESBL production by combination disk tests using cefotaxime and ceftazidime with and without clavulanic acid (Becton Dickinson, Heidelberg, Germany) according to the Clinical and Laboratory Standards Institute (CLSI) standards [92]. Furthermore, cefoxitin (30 μg) (BD, Heidelberg, Germany) was added to this test to detect AmpC phenotypes.

### 4.2. Antimicrobial and Biocide Susceptibility Testing

Antimicrobial susceptibility testing of β-lactamase-producing Enterobacterales and *Salmonella* isolates was carried out by agar disk-diffusion according to the CLSI standards [92]. *Escherichia coli* ATCC® 25922 served as the quality control strain. Disks containing the following antimicrobial agents were used: amoxicillin-clavulanate (20/10 µg), piperacillin (10 µg), cefotaxime (30 µg), ceftazidime (30 µg), cefoxitin (30 µg), aztreonam (30 µg), imipenem (10 µg), meropenem (10 µg), gentamicin (10 µg), tobramycin (10 µg), amikacin (30 µg), ciprofloxacin (5 µg), trimethoprim-sulfamethoxazole (1.25/23.75 µg), tetracycline (30 µg), doxycycline (30 µg), chloramphenicol (30 µg), and fosfomycin (200 µg) (Becton Dickinson, Heidelberg, Germany). AmpC-hyperproducing isolates (stably de-repressed) of *Enterobacter* sp. and *Citrobacter* sp. were defined as those with a cefotaxime and ceftazidime MIC of ≥32 mg/L without ESBL production, and the boronic acid inhibition test was performed by the standard disk diffusion method, using cefotaxime disks alone and in combination with 300 μg of 3-aminophenylboronic acid [93].

Biocide susceptibility testing was performed according to the previously established protocol of Schug et al. [94]. Benzalkonium chloride (Acros Organics, Geel, Belgium, 21541), representing the quaternary ammonium compounds, was tested at concentration ranges 0.000015%–0.016%, chlorhexidine (Sigma-Aldrich, Schnelldorf, Germany, 55-56-1), representing cationic compounds, was tested at concentration ranges 0.000015%–0.002%, glutardialdehyde (Chempur, Piekary Slaskie, Poland, 424610240), representing aldehydes, was tested at concentration ranges 0.0075%–1%, and isopropanol (99.9%, PHPU Eurochem BGD, Tarnow, Poland), representing alcohols, was tested at concentration ranges 1–14%. The method was performed in 96-wells U-bottom polystyrene microtiter plates (Sarstedt, Numbrecht, Germany, 82.1582.001). The bacterial inoculum was prepared according to the CLSI standards [92] using Trypticasein soy broth (Biomaximna, Lublin, Poland, PS 23–500). The final concentration of bacteria inoculated into the wells was 2.5–5 × 10^5^ CFU/mL.

### 4.3. Characterization of Genotypic Resistance

Resistance genes were analyzed by CarbDetect-AS-2 Kit microarray (Alere, Jena, Germany) [95]. Since CarbDetect-AS-2 Kit was not available to analyze all isolates, the following resistance genes were screened via PCRs: *bla*_CMY_, *bla*_CTX-M_, *bla*_OXA-1_, *bla*_OXA-2_, *bla*_SHV_, *bla*_TEM_, *sul1, sul2, sul3, dfrA1, dfrA12, dfrA14, dfrA17, dfrA19, strA, strB, aadA1, aadA2, aadA4, aadB, qepA, qnrA, qnrB, qnrC, qnrD, qnrS,* and *aac(6´)-Ib* [96,97,98,99,100,101,102,103,104,105,106,107], depending on their resistance phenotype and those genes that responded positively in the array. If resistance genes were not included in the array kit (i.e., *catA, cfr, cmlA, floR, aadA5, tet*(A), *tet*(B), *tet*(C), *tet*(D), *tet*(E), *tet*(G)), they were analyzed by PCRs as described elsewhere [104,108]. A list of all primers and PCR conditions used in this study is provided in Supplementary Table S5. Multiplex PCR for detection of AmpC genes was performed in *L. amnigena* and *Enterobacter* spp. isolates [109,110]. In addition, the genes *bla*_CMY_*, bla*_CTX-M_, *bla*_SHV_, and *bla*_TEM_ were sequenced after PCR amplification. All amplicons in the present study were sequenced at LGC Genomics, Berlin, Germany. Sequences were aligned with BLAST (https://blast.ncbi.nlm.nih.gov/Blast.cgi, accessed on 30 April 2021) and compared with reference sequences available in GenBank and the National Center for Biotechnology Information (NCBI) database (http://www.ncbi.nlm.nih.gov/pathogens/beta-lactamase-data-resources/, accessed on 30 April 2021). The quinolone resistance-determining regions (QRDR) of *gyrA* and *parC* in ciprofloxacin-resistant isolates were amplified by PCR and sequenced [111]. *E. coli* isolates displaying an AmpC phenotype were also analyzed for mutations in the chromosomal *ampC* promoter/attenuator region as described previously [112].

### 4.4. Molecular Typing Methods

The phylogroup of the *E. coli* isolates was determined by the revisited Clermont method [113]. Clonal relatedness of *E. coli* isolates was assessed by two-locus sequence typing of combined data of *fumC* and *fimH* sequences, as described by Weissman et al. [114], using CHTyper hosted at Center for Genomic Epidemiology (https://cge.cbs.dtu.dk/services/chtyper/, accessed on 30 April 2021) [115]. Allele and CH clonotype numbers were used for goeBURST analysis using PHYLOViZ [116]. Clonal relatedness of *Klebsiella pneumoniae* isolates was characterized by MLST database available at Institute Pasteur MLST (https://bigsdb.pasteur.fr/klebsiella/klebsiella.html, accessed on 30 April 2021) [117,118].

### 4.5. Virulence Genes

Detection and analysis of virulence genes was performed using primer and probe sets derived from microarray-based methods, as described previously [119,120], and used in custom made microarrays from INTER-ARRAY (INTER-ARRAY by fzmb GmbH, Bad Langensalza, Germany) according to manufacturers instructions. The list of virulence-associated genes is available at INTER-ARRAY website (https://www.inter-array.com/#microarrays, accessed on 30 April 2021) upon request.

### 4.6. Whole-Genome Sequencing (WGS)

Selected *E. coli* isolates were analyzed by WGS, which was performed by isolating bacterial DNA using the MagAttract HMW DNA Kit (Qiagen, Hilden, Germany). Ready-to-sequence libraries were prepared using Nextera XT DNA Library Preparation Kit (Illumina, San Diego, United States). Isolates were paired-end-sequenced using the Illumina MiSeq platform with a read length of 2 × 300 bp [121]. De novo assembly of raw reads was performed using SPAdes v.3.9.0 [122], and WGS data analysis was performed with SeqSphere+ software (Ridom, Münster, Germany). To assess the genetic relatedness between the *E. coli* isolates, MLST and cgMLST were performed as previously described [123]. *E. coli* phylotypes were extracted from WGS by Clermontyping (http://clermontyping.iame-research.center/, accessed on 30 April 2021) [124,125]. To identify acquired resistance genes and/or chromosomal mutations, Comprehensive Antibiotic Resistance Database (CARD; https://card.mcmaster.ca/home, accessed on 30 April 2021) [126], as well as ResFinder 4.1 (https://cge.cbs.dtu.dk/services/ResFinder/, accessed on 30 April 2021) [127,128], were used. Genes associated with biocide resistance were compared with BacMet database (Antibacterial Biocide and Metal Resistance Genes Database, http://bacmet.biomedicine.gu.se/, accessed on 30 April 2021) [129]. Virulence genes were identified using VirulenceFinder 2.0 (https://cge.cbs.dtu.dk/services/VirulenceFinder/, accessed on 30 April 2021) [130,131], CH types were characterized as mentioned above, and serogenotypes were analyzed by SerotypeFinder (https://cge.cbs.dtu.dk/services/SerotypeFinder/, accessed on 30 April 2021) [132]. The presence of plasmids was determined using PlasmidFinder 2.1 (https://cge.cbs.dtu.dk/services/PlasmidFinder/, accessed on 30 April 2021) [133]. Probability prediction of the location of a given antimicrobial resistance gene or virulence gene was achieved by applying mlplasmids trained on *E. coli* [134]. Posterior probability scores >0.7 and a minimum contig length of 1000 bp indicate that a given contig is plasmid-derived. For resistance and virulence genes with ppp scores below 0.7 but above 0.5, BLAST searches for the respective contig sequence were performed. If the BLAST search listed only plasmids for the first 30 entries with 100% coverage and identities, a plasmid location was assumed. The genomes of WGS isolates were deposited under PRJNA725684 in the NCBI BioProject database.

### 4.7. Characterization of Salmonella Isolates

The antigenic formula of all *Salmonella* isolates was determined by the National Reference Centre for *Salmonella* Austria using standard agglutination methods (ISO/TR 6579-3, 2014), and the serotype name was assigned according to the White–Kauffmann–Le Minor scheme [135].

The following virulence genes associated with *Salmonella* spp. were analyzed by PCRs: *hilA, stn,* and *tviA* [136,137].

## 5. Conclusions

In conclusion, the present study contributes to the growing evidence that β-lactamase-producing and multidrug-resistant Enterobacterales, as well as *Salmonella*, are currently part of the microbiome of wild animals. The presence of pandemic clones in samples originating from marine mammals demonstrates the complexity in the dissemination of antimicrobial drug resistance and highlights the public health threats. Furthermore, the resistance and virulence genes frequently encoded on mobile genetic elements can easily be transferred horizontally and also between different species in such a habitat and ecosystem, emphasizing the need of a One Health approach to tackling the global AMR crisis.

## Figures and Tables

**Table 1:** Characterization of ESBL-/AmpC-producing *Escherichia* (*E.*) *coli* isolated from marine mammals.

Isolate	Animal ^1^	Species	Phylo- Group	CH- Clonotype	ESBL/ AmpC	Phenotype ^2^	Genotype	Virulence Genes	*ampC*-Promoter ^3^	QRDR GyrA ^4^	QRDR ParC ^4^
**3**	Zc	*E. coli*	B1	CH65-26	AmpC	AMP, AMC, PIP, CFZ		*fimH*	39	n.d.	n.d.
**8/2**	Zc	*E. coli*	A	CH65-26	AmpC	AMP, AMC, PIP, CFZ, CTX, CAZ	*bla*_CMY-2_	*fimH*	39	n.d.	n.d.
**11/1**	Ma	*E. coli*	F	CH88-145	AmpC	AMP, AMC, PIP, CFZ	*bla*_TEM-1_, *bla*_CMY-2_, *sul2, dfrA1, aadA1, strA, strB*	*fimH, astA*	w.t.	n.d.	n.d.
**17**	Pv	*E. coli*	B2	CH103-212	AmpC	AMP, AMC, PIP, CFZ, FOF	*bla*_CMY-2_, *dfrA14, dfrA19*	*fimH, cnf1, hylA*	58	n.d.	n.d.
**21**	Zc	*E. coli*	B1	CH65-38	AmpC and ESBL	AMC, PIP, CFZ, CTX, CAZ, ATM, SXT, TET, CHL, GEN, TOB, CIP	*bla*_CTX-M-15_, *bla*_OXA-1_, *sul1, dfrA17, tet*(B)*, cmlA, aadA4, aadA5, aac(6’)-Ib*	*fimH, iucD, papC*	39	Ser83Leu, Asp87Asn	Ser80Ile
**41**	Zc	*E. coli*	B1	CH29-31	ESBL	AMC, PIP, CFZ, CTX, CAZ, SXT	*bla*_CTX-M-15_, *dfrA17, aadA4*	*fimH, astA*	n.a.	n.d.	n.d.
**53/1**	Zc	*E. coli*	E	CH148-25	AmpC	AMC, PIP, CFZ, CTX, CAZ, SXT, TET, CHL	*bla*_TEM-1_, *bla*_CMY-2_, *dfrA17, tet*(B)*, catA, strA, strB*	*fimH, iucD, papC, bfpA, cdtA, tcdAp2*	n.a.	n.d.	n.d.
**90**	Zc	*E. coli*	F	CH4-153	AmpC and ESBL	AMC, PIP, CFZ, CTX, CAZ, ATM, SXT	*bla*_CTX-M-15_, *bla*_CMY-2_, *sul2, dfrA14, strA, strB, qnrS*	*fimH*	n.a.	n.d.	n.d.
**92**	Zc	*E. coli*	E	CH148-25	AmpC	AMC, PIP, CFZ, CTX, CAZ, SXT, TET	*bla*_TEM-1_, *bla*_CMY-2_, *sul2, dfrA17, tet*(B), *aadA4, strA, strB*	*fimH, iucD, papC*	w.t.	n.d.	n.d.
**117**	Zc	*E. coli*	B2	CH103-9	AmpC	AMC, PIP, CFZ, CTX, CAZ	*bla*_CMY-2_	*fimH, cnf1*	n.a.	n.d.	n.d.
**147a**	Zc	*E. coli*	A	CH7-0	AmpC	AMC, PIP, CFZ, CTX, CAZ, ATM, TET	*bla*_TEM-1_, *bla*_CMY-2_, *dfrA5*, *tet*(A)	*fimH*	w.t.	n.d.	n.d.
**147b**	Zc	*E. coli*	B2	CH40-21	AmpC	AMC, PIP, CFZ, CTX, CAZ, SXT, TET, CHL, GEN, TOB	*bla*_TEM-1_, *bla*_CMY-2_, *sul1, sul2, dfrA1, tet*(A), *catA, cmlA, floR, aadB, strA, strB*	*fimH, astA, cnf1*	w.t.	n.d.	n.d.
**149a**	Ma	*E. coli*	A	CH7-0	AmpC	AMC, PIP, CFZ, CTX, CAZ, ATM, TET	*bla*_TEM-1_, *bla*_CMY-2_, *tet*(A)	*fimH*	n.a.	n.d.	n.d.
**156**	Zc	*E. coli*	A	CH11-0	AmpC	AMC, CFZ, CTX, CAZ, SXT, TET, CIP	*bla*_CMY-2_, *sul1, dfrA17, tet*(B), *aadA4*	*fimH, astA, iucD, papC*	w.t.	Ser83Leu, Asp87Asn	Ser80Ile
**158**	Zc	*E. coli*	E	CH148-25	AmpC	AMC, PIP, CFZ, CTX, CAZ, ATM, SXT, TET, CHL	*bla*_TEM-1_, *bla*_CMY-2_, *sul2, dfrA17, tet*(B), *catA, aadA4, strA, strB*	*fimH, iucD, papC*	w.t.	n.d.	n.d.

^1^ Abbreviations: Ma, *Mirounga angustirostris*; Pv, *Phoca vitulina*; Zc, *Zalophus californianus*. ^2^ Abbreviations: AMC, amoxicillin/clavulanate; ATM, aztreonam; CAZ, ceftazidime; CHL, chloramphenicol; CIP, ciprofloxacin; CFZ, cefazolin; CTX, cefotaxime; FOF, fosfomycin; FOX, cefoxitin; GEN, gentamicin; PIP, piperacillin; SXT, trimethoprim/sulfamethoxazole; TET, tetracycline; TOB, tobramycin. ^3^ n.a., not amplifiable; w.t., wild type. ^4^ QRDR, quinolone resistance-determining region; n.d., not done.

**Table 2:** Characterization of ESBL-/AmpC-producing Enterobacterales, other than *E. coli*, isolated from marine mammals.

Isolate	Animal ^1^	Species ^2^	ST (*Klebsiella*) ^3^	ESBL/AmpC	Phenotype ^4^	Genotype	QRDR GyrA ^5^	QRDR ParC ^5^
**8/1**	Zc	*K. pneumoniae*	466	AmpC	AMC, PIP, CFZ, CTX, CAZ, SXT, TET, CHL, GEN, TOB,	*bla*_SHV-33_, *bla*_TEM-1_, *bla*_CMY-2_, *sul1, sul2, dfrA1, tet*(A), *catA, cmlA, floR, aadB*, *strA*, *strB*	n.d.	n.d.
**10**	Zc	*K. pneumoniae*	466	AmpC	AMC, PIP, CFZ, CTX, CAZ, SXT, TET, CHL, GEN, TOB, FOF, NIT	*bla*_SHV-33_, *bla*_TEM-1_, *bla*_CMY-2_, *sul1, sul2, dfrA1, tet*(A), *catA, cmlA, floR, aadB, strA, strB*	n.d.	n.d.
**11/2**	Ma	*K. pneumoniae*	466	AmpC	AMC, PIP, CFZ, CTX, CAZ, SXT, TET, CHL, GEN, TOB, FOF, NIT	*bla*_SHV-33_, *bla*_TEM-1_, *bla*_CMY-2_, *sul1, sul2, dfrA1, tet*(A), *catA, cmlA, floR aadB, strA, strB*	n.d.	n.d.
**14**	Pv	*K. pneumoniae*	466	AmpC	AMC, PIP, CFZ, CTX, CAZ, SXT, TET, CHL, GEN, TOB, FOF, NIT	*bla*_SHV-33_, *bla*_TEM-1_, *bla*_CMY-2_*, sul1, sul2, dfrA1, tet*(A)*, catA, cmlA, floR, aadB, strA, strB*	n.d.	n.d.
**16**	Pv	*K. pneumoniae*	466	AmpC	AMC, PIP, CFZ, CTX, CAZ, SXT, TET, CHL, GEN, TOB, FOF, NIT	*bla*_SHV-33_, *bla*_TEM-1_, *bla*_CMY-2_, *sul1, dfrA1, tet*(A)*, catA, cmlA, floR, aadB, strA, strB*	n.d.	n.d.
**19**	Pv	*K. pneumoniae*	466	AmpC	AMC, PIP, CFZ, CTX, CAZ, SXT, TET, CHL, GEN, TOB, FOF, NIT	*bla*_SHV-33_, *bla*_TEM-1_, *bla*_CMY-2_, *sul1, sul2, dfrA1, tet*(A)*, catA, cmlA, floR, aadB, strA, strB*	n.d.	n.d.
**154a**	Zc	*K. pneumoniae*	466	AmpC	AMC, PIP, CFZ, CTX, CAZ, SXT, TET, CHL, GEN, TOB	*bla*_SHV-33_, *bla*_TEM-1_, *bla*_CMY-2_, *sul1, sul2, dfrA1, tet*(A), *catA, cmlA, floR, aadB, strA, strB*	n.d.	n.d.
**160**	Zc	*K. pneumoniae*	466	AmpC	AMC, PIP, CTX, CAZ, SXT, TET, CHL, GEN, TOB	*bla*_TEM-1_, *bla*_CMY-2_, *sul1, sul2, dfrA1, tet*(A), *catA, cmlA, floR, aadB, strA, strB*	n.d.	n.d.
**161**	Pv	*K. pneumoniae*	405	AmpC	AMC, PIP, SXT, TET, CHL, GEN, TOB, CIP	*bla*_SHV-11_, *bla*_TEM-1_, *bla*_OXA-1_, *bla*_CMY-2_, *sul1, sul2, dfrA1, dfrA14, tet*(A), *catA, cmlA, floR, aac(6´)-Ib, aadB, strA, strB, qnrB*	w.t.	w.t.
**164**	Zc	*K. pneumoniae*	466	AmpC	AMC, PIP, CFZ, CTX, CAZ, SXT, TET, CHL, GEN, TOB	*bla*_SHV-33_*, bla*_TEM-1_, *bla*_CMY-2_, *sul2, dfrA1, tet*(A), *catA, cmlA, floR, aadB, strA, strB*	n.d.	n.d.
**173**	Ma	*K. pneumoniae*	405	AmpC	AMC, PIP, CFZ, CTX, CAZ, SXT, TET, CHL, GEN, TOB, CIP	*bla*_SHV-11_, *bla*_TEM-1_, *bla*_OXA-1_, *bla*_CMY-2_, *sul1, sul2, dfrA1, dfrA14, tet*(A), *catA, cmlA, floR, aac(6′)-Ib, aadB, strA, strB, qnrB*	w.t.	n.a.
**175**	Zc	*K. pneumoniae*	405	AmpC	AMC, PIP, CFZ, CTX, CAZ, SXT, TET, CHL, GEN, TOB, CIP	*bla*_SHV-11_, *bla*_TEM-1_, *bla*_OXA-1_, *bla*_CMY-2_, *sul1, sul2, dfrA1, dfrA14, tet*(A), *catA, cmlA, floR, aac(6′)-Ib, aadB, strA, strB, qnrB*	w.t.	n.a.
**9**	Zc	*C. koseri*	n.a.	AmpC de-repression	intrinsic + CTX, CAZ, TET, CHL	*bla*_CMY_, *sul2, tet*(A), *cmlA, floR, strA, strB*	n.d.	n.d.
**28**	Zc	*C. koseri*	n.a.	AmpC de-repression	intrinsic + CTX, CAZ, SXT, TET, CHL, GEN, TOB, FOF, CIP	*bla*_TEM-1_, *bla*_OXA-1_, *bla*_CMY_, *sul1, sul2, dfrA1, dfrA14, tet*(A)*, cmlA, aadB, aac(6′)-Ib, strA, strB, qnrB*	w.t.	w.t.
**30**	Zc	*C. koseri*	n.a.	AmpC de-repression	intrinsic + CTX, CAZ, SXT	*bla*_CMY_, *sul2*	w.t.	n.a.
**35**	Zc	*C. koseri*	n.a.	AmpC de-repression	intrinsic + CTX, CAZ, SXT, TET, CHL, GEN, TOB, FOF, CIP	*bla*_TEM-1_, *bla*_OXA-1_, *bla*_CMY_, *sul1, sul2, dfrA1, dfrA14, tet*(A)*, cmlA, aadB, aac(6´)-Ib, strA, strB, qnrB*	w.t.	w.t.
**53/2**	Zc	*C. koseri*	n.a.	AmpC de-repression	intrinsic + CTX, CAZ, SXT, TET, CHL	*bla*_TEM-1_, *bla*_CMY_, *sul2, dfrA14*, *tet*(A), *tet*(B), *catA, cmlA, floR, strA, strB, qnrB*	n.d.	n.d.
**149b**	Ma	*C. freundii* complex	n.a.	AmpC de-repression	AMC, PIP, CFZ, CTX, CAZ, SXT, TET, CHL, GEN, TOB	*bla*_TEM-1_, *bla*_CMY_, *sul1, dfrA12, dfrA19, tet*(A)*, aadA2, aac(6´)-IIc, strA, strB*	n.d.	n.d.
**151**	Zc	*C. freundii* complex	n.a.	AmpC de-repression	AMC, PIP, CFZ, CTX, CAZ, SXT, TET, CHL, GEN	*bla*_TEM-1_, *bla*_CMY_, *sul1, dfrA12, dfrA19, tet*(A), *aadA2, aac(6´)-IIc, strA, strB*	n.d.	n.d.
**154b**	Zc	*C. freundii* complex	n.a.	AmpC de-repression	AMC, PIP, CFZ, CTX, CAZ, SXT, TET, CHL, GEN	*bla*_TEM-1_, *bla*_CMY_, *sul1, sul2, dfrA12, dfrA19, tet*(A), *aadA2, aac(6´)-IIc, strA, strB*	n.d.	n.d.
**163**	Zc	*C. freundii* complex	n.a.	AmpC de-repression	AMC, PIP, CFZ, CTX, CAZ, SXT, TET, CHL, GEN, TOB, CIP	*bla*_TEM-1_, *bla*_CMY_, *sul1, sul2, dfrA12, dfrA19, tet*(A), *aadA2, strA, strB, qnrB*	n.a.	n.a.
**165**	Zc	*C. freundii* complex	n.a.	AmpC de-repression	AMC, PIP, CFZ, CTX, CAZ, SXT, TET, CHL, GEN, TOB	*bla*_TEM-1_, *bla*_CMY_, *sul1, sul2, dfrA12, dfrA19, tet*(A), *aadA2, strA, strB*	n.d.	n.d.
**169**	Zc	*C. koseri*	n.a.	AmpC de-repression	AMC, PIP, CFZ, CTX, CAZ, SXT, TET, GEN	*bla*_TEM-1_, *bla*_CMY_, *sul2, dfrA14, tet*(A), *strA, strB*	n.d.	n.d.
**179**	Zc	*C. koseri*	n.a.	AmpC de-repression	AMC, PIP, CFZ, CTX, CAZ, SXT, TET, GEN	*bla*_TEM-1_, *bla*_CMY_, *sul2, dfrA14, tet*(A), *strA, strB*	n.d.	n.d.
**192/1**	Zc	*C. freundii* complex	n.a.	AmpC de-repression	AMC, PIP, FOX, CFZ, CAZ, CTX, SXT, TET	*bla*_TEM-1_, *bla*_CMY_, *sul1, sul2, dfrA12, tet*(A)	n.d.	n.d.
**213**	Kb	*C. freundii* complex	n.a.	AmpC de-repression	AMC, PIP, FOX, CFZ, CTX, CAZ, SXT, TET	*bla*_CMY_*, sul1, dfrA12, tet*(A)	n.d.	n.d.
**215**	Kb	*C. freundii* complex	n.a.	AmpC de-repression	AMC, PIP, FOX, CFZ, CTX, CAZ, SXT	*bla*_CMY_, *sul1, dfrA12*	n.d.	n.d.
**224b**	Ma	*C. freundii* complex	n.a.	AmpC de-repression	AMC, PIP, FOX, CFZ, CTX, CAZ, SXT, TET, CHL, GEN, TOB	*bla*_TEM-1_, *bla*_CMY_, *sul1, sul2, dfrA1, cmlA, floR, aadB, strA, strB*	n.d.	n.d.
**226**	Zc	*C. koseri*	n.a.	AmpC de-repression	AMC, PIP, FOX, CFZ, CTX, CAZ, SXT, TET, CHL, GEN, TOB	*bla*_CMY_, *sul2, tet*(A), *tet*(D), *floR*, *strA, strB*	n.d.	n.d.
**230c**	Zc	*C. koseri*	n.a.	AmpC de-repression	AMC, PIP, FOX, CFZ, CTX, CAZ, SXT, TET, GEN	*bla*_TEM-1_, *bla*_CMY_, *sul2, dfrA14, tet*(D), *strA, strB*	n.d.	n.d.
**43**	Zc	*En. cloacae* complex	n.a.	AmpC de-repression	intrinsic + CTX, CAZ, TET, SXT	*bla*_TEM-1_, *sul2*, *dfrA14, tet*(D), *strA*, *strB*	n.d.	n.d.
**66**	Zc	*En. cancerogenus*	n.a.	AmpC de-repression	intrinsic + CTX, CAZ		n.d.	n.d.
**130**	Ma	*L. amnigena*	n.a.	AmpC and ESBL	AMC, PIP, CFZ, CTX, CAZ, SXT, CHL	*bla*_DHA-1_*, bla*_SHV-12_, *sul1, sul2, dfrA14, cmlA, floR, aac(6´)-Ib, qnrB*	n.d.	n.d.
**132**	Ma	*L. amnigena*	n.a.	AmpC and ESBL	AMC, PIP, CFZ, CTX, CAZ, SXT, CHL	*bla*_DHA-1_*, bla*_SHV-12_, *sul1, sul2, dfrA14, cmlA, floR, aac(6´)-Ib, qnrB*	n.d.	n.d.
**197/2**	Zc	*P. mirabilis*	n.a.	AmpC	AMC, PIP, CAZ, CTX, SXT, CHL, GEN	*bla*_TEM-1_, *bla*_CMY-2_, *sul1, sul2, dfrA1, catA, cmlA, floR, aadB, strA, strB*	n.d.	n.d.

^1^ Abbreviations: Kb, Kogia breviceps; Ma, Mirounga angustirostris; Pv, Phoca vitulina; Zc, Zalophus californianus. ^2^ Abbreviations: C., Citrobacter; En., Enterobacter; K., Klebsiella; L., Lelliottia; P., Proteus. ^3^ n.a., not applicable. ^4^ Abbreviations: AMC, amoxicillin/clavulanate; ATM, aztreonam; CAZ, ceftazidime; CHL, chloramphenicol; CIP, ciprofloxacin; CFZ, cefazolin; CTX, cefotaxime; FOF, fosfomycin; FOX, cefoxitin; GEN, gentamicin; PIP, piperacillin; SXT, trimethoprim/sulfamethoxazole; TET, tetracycline; TOB, tobramycin. ^5^ QRDR: quinolone resistance-determining region; n.a., not amplifiable; n.d., not done; w.t., wild type.

**Table 3:** Characterization of ESBL/AmpC-producing *E. coli* isolated from marine mammals via whole-genome sequencing.

Isolate	Animal ^1^	Phylo-Group	CH-Clono-Typing	SEROGENOTYPE ^2^	ST	ESBL/AmpC	Phenotype ^3^	Genotype	Virulence Genes	*ampC* Promoter ^4^	QRDR ^5^ GyrA ^4^	QRDR ParC ^4^	ParE ^4^
**68**	Zc	A	CH11-0	O_NT_:H9	167	ESBL	AMC, PIP, CFZ, CTX, CAZ, ATM, SXT, TET, GEN, TOB, CIP	*bla*_CTX-M-15_, *bla*_OXA-1_, *sul1, dfrA17, tet*(A), *tet*(B), *catB3, aadA4, aadA5, aac(3)-IIa, aac(6′)-Ib-cr, mdfA*	*astA, capU, fyuA, irp2, iss, iucC, iut*A*, sit*A*, ter*C	w.t.	Ser83Leu, Asp87Asn	Ser80Ile	S458A
**171**	Pv	D	CH50-299	O_NT_:H1	5748	AmpC	AMC, PIP, CFZ, CTX, CAZ	*bla*_CMY-4_, *mdfA*	*air, chuA, cia, eilA, lpfA, terC, traT*	w.t.	w.t.	w.t.	w.t.
**183**	Zc	A	CH11-0	O_NT_:H9	167	ESBL	AMC, PIP, CFZ, CTX, CAZ, ATM, SXT, TET, TOB, CIP	*bla*_CTX-M-15_, *sul1, sul2, dfrA17, tet*(A), *tet*(B), *catB3, aadA5, aph(3″)-Ib, aph(6)-Id, aac(6′)-Ib-cr, mdfA*	*astA, capU, fyuA, irp2, iss, iucC, iutA, sitA, terC, traT*	w.t.	Ser83Leu, Asp87Asn	Ser80Ile	S458A
**192/2**	Zc	B1	CH65-32	O9:H10	1431	AmpC	AMC, PIP, FOX, CFZ, CAZ, CTX, SXT, CIP	*bla*_TEM-1B_*, bla*_CMY-2_, *sul2, dfrA5, aph(3″)-Ib, aph(6)-Id, mdfA*	*capU, cba, cia, cma, cvaC, etsC, fyuA, hlyF, iroN, irp2, iss, iucC, iutA, lpfA, mchF, ompT, sitA, terC, traT*	-19	Ser83Leu, Asp87Asn	Ser80Ile	S458A
**197/1**	Zc	F	CH4-58	O_NT_:H42	648	AmpC	AMC, PIP, FOX, CFZ, CAZ, CTX, ATM, SXT, TET, CHL, GEN, TOB	*bla*_TEM-1B_, *bla*_CMY-2_, *sul1, sul2, dfrA1, tet*(A), *cmlA1, floR, aph(3″)-Ib, aph(6)-Id, ant(2″)-Ia, mdfA*	*air, cea, celb, chuA, focCsfaE, focG, fyuA, iroN, irp2, kpsE, kpsMII, lpfA, mchB, mchC, mchF, mcmA, sfaD, sitA, terC, yfcV*	-28	w.t.	w.t.	w.t.
**202**	Zc	B2	CH103-9	O_NT_:H31	372	AmpC	AMC, PIP, FOX, CFZ	*mdfA*	*cea, chuA, cnf1, focCsfaE, focG, focI, fyuA, hra, ibeA, iroN, irp2, iss, mchB, mchC, mchF, mcmA, ompT, papA, papC, sitA, terC, usp, vat, yfcV*	-32	w.t.	w.t.	w.t.
**206**	Zc	D	CH26-26	O_NT_:H18	963	AmpC	AMC, PIP, FOX, CFZ, CTX, CAZ	*bla*_CMY-2_, *mdfA*	*air, astA, chuA, eilA, fyuA, irp2, kpsE, senB, sitA, terC, traT*	w.t.	w.t.	w.t.	w.t.
**209**	Zc	D	CH26-0	O_NT_:H15	38	ESBL	AMC, PIP, FOX, CFZ, CTX, CAZ	*bla*_CTX-M-15_, *bla*_TEM-1B_, *qnrS1, mdfA*	*air, chuA, eilA, iss, kpsE, kpsMII, terC*	w.t.	w.t.	w.t.	w.t.
**224a**	Ma	F	CH37-1572 *	O_NT_:H28	4957	AmpC	AMC, PIP, FOX, CFZ, CTX, CAZ, SXT, TET, CHL, GEN, TOB	*bla*_TEM-1B_, *bla*_CMY-2_, *sul1, sul2, dfrA1, tet*(A), *cmlA1, floR, aph(3″)-Ib, aph(6)-Id, ant(2″)-Ia, mdfA*	*air, chuA, cia, cvaC, etsC, hlyF, iroN, iss, lpfA, mchF, ompT, sitA, terC, traT, usp, yfcV*	-28	w.t.	w.t.	w.t.
**230a**	Zc	D	CH36-93	O_NT_:H15	349	AmpC	AMC, FOX, CFZ, CAZ	*bla*_CMY-2_, *mdfA*	*cba, chuA, cia, cma, eilA, fyuA, hra, irp2, kpsE, terC, traT*	w.t.	w.t.	w.t.	S458A
**230b**	Zc	A	CH11-0	O_NT_:H9	167	AmpC	AMC, FOX, CFZ, CTX, CAZ, SXT, TET, FOF, CIP	*bla*_CMY-2_, *sul1, dfrA17, tet*(A), *tet*(B), *aadA5, mdfA*	*astA, capU, cba, cia, cma, fyuA, irp2, iss, iucC, iutA, sitA, terC, traT*	w.t.	w.t.	Ser80Ile	w.t.
**234**	Zc	A	CH11-54	O_NT_:H4	484	ESBL	AMP, CFZ, CTX	*bla*_CTX-M-15_, *qnrS1, mdfA*	*aap, astA, iss, kpsE, kpsMII, ompT, terC, traT*	w.t.	w.t.	w.t.	I355T

^1^ Abbreviations: Ma, *Mirounga angustirostris*; Pv, *Phoca vitulina*; Zc, *Zalophus californianus*. ^2^ _NT_, not typeable. ^3^ Abbreviations: AMC, amoxicillin/clavulanate; ATM, aztreonam; CAZ, ceftazidime; CHL, chloramphenicol; CIP, ciprofloxacin; CFZ, cefazolin; CTX, cefotaxime; FOF, fosfomycin; FOX, cefoxitin; GEN, gentamicin; PIP, piperacillin; SXT, trimethoprim/sulfamethoxazole; TET, tetracycline; TOB, tobramycin. ^4^ w.t., wild type. ^5^ QRDR: quinolone resistance-determining region. * new allele.

**Table 4:** Characterization of *Salmonella* isolated from marine mammals.

Isolate	Animal ^1^	Serotype ^2^	Phenotype ^3^	Genotype	Virulence Genes
8. S.	Zc	*S*. Saintpaul (antigenic formula: 1,4,5,12 : e,h : 1,2)	n.r.		*hilA, stn*
27 S.	Zc	*S*. Newport (antigenic formula 6,8 : e,h : 1,2)	n.r.		*hilA, stn*
42 S.	Zc	AmpC *S*. Saintpaul (antigenic formula 1,4,5,12 : e,h : 1,2)	AMC, PIP, CFZ, CTX, CAZ	*bla*_CMY-2_	*hilA, stn*
56 S.	Zc	*S*. Havana (antigenic formula 1,13,23 : f,g : -)	n.r.		*hilA, stn*
62 S.	Zc	*S*. Havana (antigenic formula 1,13,23 : f,g : -)	n.r.		*hilA, stn*
91 S.	Zc	*S*. Havana (antigenic formula 1,13,23 : f,g : -)	n.r.		*hilA, stn*
113 S.	Zc	*S*. Havana (antigenic formula 1,13,23 : f,g : -)	n.r.		*hilA, stn*
115 S.	Ma	*S*. Havana (antigenic formula 1,13,23 : f,g : -)	n.r.		*hilA, stn*
125 S.	Ma	*S*. Newport (antigenic formula 6,8 : e,h : 1,2)	n.r.		*hilA, stn*
127 S.	Ma	*S*. Havana (antigenic formula 1,13,23 : f,g : -)	n.r.		*hilA, stn*
135 S.	Ma	*S*. Reading (antigenic formula 1,4,5,12 : e,h : 1,5)	n.r.		*hilA, stn*
226 S.	Zc	*S*. Braenderup (antigenic formula 6,7 : e,h : e,n,z15)	n.r.		*hilA, stn*
245 S.	Zc	*S*. Reading (antigenic formula 1,4,5,12 : e,h : 1,5)	n.r.		*hilA, stn*
255 S.	Zc	*S*. Reading (antigenic formula 1,4,5,12 : e,h . 1,5)	n.r.		*hilA, stn*
273 S.	Zc	*S*. Albany (antigenic formula 8,20 : z4,z24 : -)	n.r.		*hilA, stn*
277 S.	Ma	*S*. Albany (antigenic formula 8,20 : z4,z24 : -)	n.r.		*hilA, stn*
279 S.	Ma	*S*. Havana (antigenic formula 1,13,23 : f,g : -)	n.r.		*hilA, stn*
281 S.	Zc	*S*. Havana (antigenic formula 1,13,23 : f,g : -)	n.r.		*hilA, stn*

^1^ Abbreviations: Ma, *Mirounga angustirostris*; Zc, *Zalophus californianus*. ^2^ *S*., *Salmonella*. ^3^ Abbreviations: n.r., non-resistant; AMC, amoxicillin/clavulanate; CAZ, ceftazidime; CFZ, cefazolin; CTX, cefotaxime; PIP, piperacillin.

## Data Availability

All data is contained within the article or supplementary material.

## Supplementary Materials

The following are available online at https://www.mdpi.com/article/10.3390/ijms22115905/s1.
