# Supplementary material for: Presence of β-Lactamase-producing Enterobacterales and Salmonella Isolates in Marine Mammals
Source: Int J Mol Sci. 2021 May 31;22(11):5905. doi: 10.3390/ijms22115905 (PMC8199236; doi:10.3390/ijms22115905)
Supplement: Supplementary file 1 [file ijms-22-05905-s001.zip › Table S5_List of primers FINAL.pdf]

Supplementary Table S5. List of primers used in this study

Name of primer	Primer sequence (5´-3´)	Target	Ta (°C)	Amplicon size (bp)	Reference*
CMY /F	ATG ATG AAA AAA TCG TTA TGC TGC	bla_{CMY}	58	1138	[107]
CMY /R	GCT TTT CAA GAA TGC GCC AGG				
CTX-M /F	ATG TGC AGY ACC AGT AAR GTK ATG GC	$bla_{\text{CTX-M}}$	55	592	[107]
CTX-M /R	TGG GTR AAR TAR GTS ACC AGA AYS AGC GG				
OXA-1 /F	ATG AAA AAC ACA ATA CAT ATC AAC TTC GC	$bla_{\text{OXA-1}}$	55	820	[107]
OXA-1 /R	GTG TGT TTA GAA TGG TGA TCG CAT T				
OXA-2 /F	ACG ATA GTT GTG GCA GAC GAA C	$bla_{\text{OXA-2}}$	55	601	[107]
OXA-2 /R	ATY CTG TTT GGC GTA TCR ATA TTC				
SHV /F	TTA TCT CCC TGT TAG CCA CC	bla_{SHV}	55	796	[107]
SHV /R	GAT TTG CTG ATT TCG CTC GG				
TEM /F	GCG GAA CCC CTA TTT G	bla_{TEM}	55	964	[107]
TEM /R	ACC ATT GCT TAA TCA GTG AG				
MOXMF	GCT GCT CAA GGA GCA CAG GAT	$bla_{\text{MOX-1}}$, $bla_{\text{MOX-2}}$, $bla_{\text{CMY-1}}$, $bla_{\text{CMY-8}}$	64	520	[110]
MOXMR	CAC ATT GAC ATA GGT GTG GTG C	to $bla_{\text{CMY-11}}$			
CITMF	TGG CCA GAA CTG ACA GGC AAA	$bla_{\text{LAT-1}}$ to $bla_{\text{LAT-4}}$, $bla_{\text{CMY-2}}$ to	64	462	[110]
CITMR	TTT CTC CTG AAC GTG GCT GGC	$bla_{\text{CMY-7}}$, $bla_{\text{BIL-1}}$			
DHAMF	AAC TTT CAC AGG TGT GCT GGG T	$bla_{\text{DHA-1}}$, $bla_{\text{DHA-2}}$	64	405	[110]
DHAMR	CCG TAC GCA TAC TGG CTT TGC				
ACCMF	AAC AGC CTC AGC AGC CGG TTA	bla_{ACC}	64	346	[110]
ACCMR	TTC GCC GCA ATC ATC CCT AGC				
EBCMF	TCG GTA AAG CCG ATG TTG CGG	$bla_{\text{MIR-1}}$, $bla_{\text{ACT-1}}$	64	302	[110]
EBCMR	CTT CCA CTG CGG CTG CCA GTT				
FOXMF	AAC ATG GGG TAT CAG GGA GAT G	$bla_{\text{FOX-1}}$ to $bla_{\text{FOX-5b}}$	64	190	[110]
FOXMR	CAA AGC GCG TAA CCG GAT TGG				

MultiCaseACC_for<br>MultiCaseACC_rev	CAC CTC CAG CGA CTT GTT AC<br>GTT AGC CAG CAT CAC GAT CC	*bla*$_{ACC-1}$, *bla*$_{ACC-2}$	60	346	[109]
MultiCaseFOX_for<br>MultiCaseFOX_rev	CTA CAG TGC GGG TGG TTT<br>CTA TTT GCG GCC AGG TGA	*bla*$_{FOX-1}$ to *bla*$_{FOX-5}$	60	162	[109]
MultiCaseMOX_for<br>MultiCaseMOX_rev	GCA ACA ACG ACA ATC CAT CCT<br>GGG ATA GGC GTA ACT CTC CCA A	*bla*$_{MOX-1}$, *bla*$_{MOX-2}$, *bla*$_{CMY-1}$, *bla*$_{CMY-8}$ to *bla*$_{CMY-11}$, *bla*$_{CMY-19}$	60	895	[109]
MultiCaseDHA_for<br>MultiCaseDHA_rev	TGA TGG CAC AGC AGG ATA TTC<br>GCT TTG ACT CTT TCG GTA TTC G	*bla*$_{DHA-1}$, *bla*$_{DHA-2}$	60	997	[109]
MultiCaseCIT_for<br>MultiCaseCIT_rev	CGA AGA GGC AAT GAC CAG AC<br>ACG GAC AGG GTT AGG ATA GY	*bla*$_{LAT-1}$ to *bla*$_{LAT-3}$, *bla*$_{BIL-1}$, *bla*$_{CMY-2}$ to *bla*$_{CMY-7}$, *bla*$_{CMY-12}$ to *bla*$_{CMY-18}$, *bla*$_{CMY-21}$ to *bla*$_{CMY-23}$	60	538	[109]
MultiCaseEBC_for<br>MultiCaseEBC_rev	CGG TAA AGC CGA TGT TGC G<br>AGC CTA ACC CCT GAT ACA	*bla*$_{ACT-1}$, *bla*$_{MIR-1}$	60	683	[109]
sul1 /F<br>sul1 /R	CTT CGA TGA GAG CCG GCG GC<br>GCA AGG CGG AAA CCC GCG CC	*sul1*	68	417	[104]
sul2 /F<br>sul2 /R	AGG GGG CAG ATG TGA TCG AC<br>GCA GAT GAT TTC GCC AAT TG	*sul2*	58	249	[104]
sul3 /F<br>sul3 /R	GAG CAA GAT TTT TGG AAT CG<br>CAT CTG CAG CTA ACC TAG GGC TTT GGA	*sul3*	55	789	[104]
dfrA1 /F<br>dfrA1 /R	ACG GAT CCT GGC TGT TGG TTG GAC GC<br>CGG AAT TCA CCT TCC GGC TCG ATG TC	*dfrA1*	55	254	[104]
dfrA12 /F<br>drfA12 /R	ACT CGG AAT CAG TAC GCA<br>GTG TAC GGA ATT ACA GCT	*dfrA12*	51	462	[104]
dfrA14 /F<br>dfrA14 /R	TTA ACC CAG GAT GAG AAC CT<br>CGA TTG CAT AGC TTT GTT AA	*dfrA14*	56	510	[105]
dfrA17 /F<br>dfrA17 /R	GAT TTC TGC AGT GTC AGA<br>CTC AGG CAT TAT AGG GAA	*dfrA17*	50	384	[104]
dfrA19 /F<br>dfrA19 /R	AGT CGC TGT GGA TTC TAA GT<br>CAA TGT GAA AAT TGT TCT GG	*dfrA19*	56	455	[103]
tetA /F<br>tetA /R	GCT ACA TCC TGC TTG CCT TC<br>CAT AGA TCG CCG TGA AGA GG	*tet*(A)	55	210	[104]
tetB /F<br>tetB /R	TTG GTT AGG GGC AAG TTT TG<br>GTA ATG GGC CAA TAA CAC CG	*tet*(B)	55	659	[104]
tetC /F<br>tetC /R	CTT GAG AGC CTT CAA CCC AG<br>ATG GTC GTC ATC TAC CTG CC	*tet*(C)	55	418	[104]

tetD /F<br>tetD /R	AAA CCA TTA CGG CAT TCT GC<br>GAC CGG ATA CAC CAT CCA TC	*tet*(D)	55	787	[104]
tetE /F<br>tetE /R	AAA CCA CAT CCT CCA TAC GC<br>AAA TAG GCC ACA ACC GTC AG	*tet*(E)	55	278	[104]
tetG /F<br>tetG /R	CAG CTT TCG GAT TCT TAC GG<br>GAT TGG TGA GGC TCG TTA GC	*tet*(G)	55	468	[104]
catA /F<br>catA /R	CCT GCC ACT CAT CGC AGT<br>CCA CCG TTG ATA TAT CCC	*catA*	55	623	[104]
cmlA /F<br>cmlA /R	TGT CAT TTA CGG CAT ACT CG<br>ATC AGG CAT CCC ATT CCC AT	*cmlA*	55	455	[104]
cfr /F<br>cfr /R	TGA AGT ATA AAG CAG GTT GGG AGT CA<br>ACC ATA TAA TTG ACC ACA AGC AGC	*cfr*	48	746	[108]
floR /F<br>floR /R	GCG ATA TTC ATT ACT TTG GC<br>TAG GAT GAA GGT GAG GAA TG	*floR*	50	425	[104]
aadA1 /F<br>aadA1 /R	CGA CTC AAC TAT CAG AGG TA<br>CTT TTG TCA GCA AGA TAG CC	*aadA1*	51	384	[104]
aadA2 /F<br>aadA2 /R	CGG TGA CCA TCG AAA TTT CG<br>CTA TAG CGC GGA GCG TCT CGC	*aadA2*	55	249	[104]
aadA4 /F<br>aadA4 /R	ATC TTG CGA TTT TGC TGA CC<br>TGT ACC AAA TGC GAG CAA GA	*aadA4*	53	198	[102]
aadA5 /F<br>aadA5 /R	CAC TGG ACA CAA TCC ACC TG<br>CCA AGG CAC TAC TTC GCT TC	*aadA5*	55	217	[104]
aadB /F<br>aadB /R	GGG CGC GTC ATG GAG GAG TT<br>TAT CGC GAC CTG AAA GCG GC	*aadB*	67	328	[96]
strA /F<br>strA /R	CCT ATC GGT TGA TCA ATG TC<br>GAA GAG TTT TAG GGT CCA CC	*strA*	58	250	[104]
strB /F<br>strB /R	ATC GTC AAG GGA TTG AAA CC<br>GGA TCG TAG AAC ATA TTG GC	*strB*	55	509	[97]
qepA /F<br>qepA /R	GCA GGT CCA GCA GCG GGT AG<br>CTT CCT GCC CGA GTA TCG TG	*qepA*	60	199	[100]
qnrA /F<br>qnrA /R	ATT TCT CAC GCC AGG ATT TG<br>GAT CGG CAA AGG TTA GGT CA	*qnrA*	53	516	[98]
qnrB /F<br>qnrB /R	GAT CGT GAA AGC CAG AAA GG<br>ACG ATG CCT GGT AGT TGT CC	*qnrB*	53	469	[98]
qnrC /F<br>qnrC /R	GGG TTG TAC ATT TAT TGA ATC<br>TCC ACT TTA CGA GGT TCT	*qnrC*	50	447	[106]

qnrD /F<br>qnrD /R	CGA GAT CAA TTT ACG GGG AAT A<br>AAC AAG CTG AAG CGC CTG	*qnrD*	50	582	[101]
qnrS /F<br>qnrS /R	ACG ACA TTC GTC AAC TGC AA<br>TAA ATT GGC ACC CTG TAG GC	*qnrS*	53	417	[98]
aac(6´)-Ib /F<br>aac(6´)-Ib /R	TTG CGA TGC TCT ATG AGT GGC TA<br>CTC GAA TGC CTG GCG TGT TT	*aac(6´)-Ib*	55	482	[99]

*Reference numbers correspond to the reference numbers in the main manuscript

References

107. Dierikx, C.M.; van Duijkeren, E.; Schoormans, A.H.W.; van Essen-Zandbergen, A.; Veldman, K.; Kant, A.; Huijsdens, X.W.; van der Zwaluw, K.; Wagenaar, J.A.; Mevius, D.J. Occurrence and characteristics of extended-spectrum-β-lactamase- and AmpC-producing clinical isolates derived from companion animals and horses. *J. Antimicrob. Chemother.* **2012**, *67*, 1368–1374, doi:10.1093/jac/dks049.

110. Pérez-Pérez, F.J.; Hanson, N.D. Detection of plasmid-mediated AmpC beta-lactamase genes in clinical isolates by using multiplex PCR. *J. Clin. Microbiol.* **2002**, *40*, 2153–2162, doi:10.1128/jcm.40.6.2153-2162.2002.

109. Dallenne, C.; Da Costa, A.; Decré, D.; Favier, C.; Arlet, G. Development of a set of multiplex PCR assays for the detection of genes encoding important beta-lactamases in *Enterobacteriaceae*. *J. Antimicrob. Chemother.* **2010**, *65*, 490–495, doi:10.1093/jac/dkp498.

104. Dolejska, M.; Frolkova, P.; Florek, M.; Jamborova, I.; Purgertova, M.; Kutilova, I.; Cizek, A.; Guenther, S.; Literak, I. CTX-M-15-producing *Escherichia coli* clone B2-O25b-ST131 and *Klebsiella* spp. isolates in municipal wastewater treatment plant effluents. *J. Antimicrob. Chemother.* **2011**, *66*, 2784–2790, doi:10.1093/jac/dkr363.

105. Miranda, A.; Ávila, B.; Díaz, P.; Rivas, L.; Bravo, K.; Astudillo, J.; Bueno, C.; Ulloa, M.T.; Hermosilla, G.; Del Canto, F.; et al. Emergence of Plasmid-Borne *dfrA14* Trimethoprim Resistance Gene in *Shigella sonnei*. *Front. Cell. Infect. Microbiol.* **2016**, *6*, 77, doi:10.3389/fcimb.2016.00077.

103. Šeputienė, V.; Povilonis, J.; Ružauskas, M.; Pavilonis, A.; Sužiedėlienė, E. Prevalence of trimethoprim resistance genes in *Escherichia coli* isolates of human and animal origin in Lithuania. *J. Med. Microbiol.* **2010**, *59*, 315–322, doi:10.1099/jmm.0.015008-0.

108. Kehrenberg, C.; Schwarz, S. Distribution of florfenicol resistance genes *fexA* and *cfr* among chloramphenicol-resistant *Staphylococcus* isolates. *Antimicrob. Agents Chemother.* **2006**, *50*, 1156–1163, doi:10.1128/AAC.50.4.1156-1163.2006.

102. Szczepanowski, R.; Linke, B.; Krahn, I.; Gartemann, K.-H.; Gützkow, T.; Eichler, W.; Pühler, A.; Schlüter, A. Detection of 140 clinically relevant antibiotic-resistance genes in the plasmid metagenome of wastewater treatment plant bacteria showing reduced susceptibility to selected antibiotics. *Microbiology (Reading)* **2009**, *155*, 2306–2319, doi:10.1099/mic.0.028233-0.

96. Sandvang, D.; Aarestrup, F.M. Characterization of aminoglycoside resistance genes and class 1 integrons in porcine and bovine gentamicin-resistant *Escherichia coli*. *Microb. Drug Resist.* **2000**, *6*, 19–27, doi:10.1089/mdr.2000.6.19.

97. Boerlin, P.; Travis, R.; Gyles, C.L.; Reid-Smith, R.; Janecko, N.; Lim, H.; Nicholson, V.; McEwen, S.A.; Friendship, R.; Archambault, M. Antimicrobial resistance and virulence genes of *Escherichia coli* isolates from swine in Ontario. *Appl. Environ. Microbiol.* **2005**, *71*, 6753–6761, doi:10.1128/AEM.71.11.6753-6761.2005.

100. Yamane, K.; Wachino, J.; Suzuki, S.; Arakawa, Y. Plasmid-mediated *qepA* gene among *Escherichia coli* clinical isolates from Japan. *Antimicrob. Agents Chemother.* **2008**, *52*, 1564–1566, doi:10.1128/AAC.01137-07.

98. Gay, K.; Robicsek, A.; Strahilevitz, J.; Park, C.H.; Jacoby, G.; Barrett, T.J.; Medalla, F.; Chiller, T.M.; Hooper, D.C. Plasmid-mediated quinolone resistance in non-Typhi serotypes of *Salmonella enterica*. *Clin. Infect. Dis.* **2006**, *43*, 297–304, doi:10.1086/505397.

106. Wang, M.; Guo, Q.; Xu, X.; Wang, X.; Ye, X.; Wu, S.; Hooper, D.C.; Wang, M. New plasmid-mediated quinolone resistance gene, *qnrC*, found in a clinical isolate of *Proteus mirabilis*. *Antimicrob. Agents Chemother.* **2009**, *53*, 1892–1897, doi:10.1128/AAC.01400-08.

101. Cavaco, L.M.; Hasman, H.; Xia, S.; Aarestrup, F.M. *qnrD*, a novel gene conferring transferable quinolone resistance in *Salmonella enterica* serovar Kentucky and Bovismorbificans strains of human origin. *Antimicrob. Agents Chemother.* **2009**, *53*, 603–608, doi:10.1128/AAC.00997-08.

99. Park, C.H.; Robicsek, A.; Jacoby, G.A.; Sahm, D.; Hooper, D.C. Prevalence in the United States of *aac(6′)-Ib-cr* encoding a ciprofloxacin-modifying enzyme. *Antimicrob. Agents Chemother.* **2006**, *50*, 3953–3955, doi:10.1128/AAC.00915-06.
